# Improving outcomes in MASLD: the role of *Helicobacter pylori* eradication and lifestyle interventions–author's response to Zhang

**DOI:** 10.1016/j.lana.2025.101080

**Published:** 2025-04-02

**Authors:** Christian S. Alvarez, M. Constanza Camargo, M. Larissa Avilés-Santa, Olga Garcia-Bedoya, Maria S. Pattany, Bharat Thyagarajan, Barry I. Graubard, Katherine A. McGlynn

**Affiliations:** a Division of Intramural Research, National Institute on Minority Health and Health Disparities, NIH, Rockville, MD, USA; b Division of Cancer Epidemiology and Genetics, National Cancer Institute, Rockville, MD, USA; c Division of Clinical and Health Services Research, National Institute on Minority Health and Health Disparities, Rockville, MD, USA; d Institute for Minority Health Research, University of Illinois Chicago, Chicago, IL, USA; e Academic Internal Medicine and Geriatrics, Department of Medicine, University of Illinois Chicago, Chicago, IL, USA; f Department of Psychology, College of Arts and Sciences, University of Miami, Coral Gables, FL, USA; g Department of Laboratory Medicine and Pathology, University of Minnesota, Minneapolis, MN, USA

We thank Dr. Zhang for the interest in our study^1^ and for noting that the study provides valuable insights into the association between *Helicobacter pylori* and metabolic dysfunction-associated steatotic liver disease (MASLD) and related conditions. We would like to take the opportunity to address several issues raised by Dr. Zhang.

Dr. Zhang highlights the importance of including additional confounders such as dietary habits, physical activity levels, socioeconomic status (SES) and health behaviors, which could influence MASLD and obesity risk. Please note that we adjusted for SES-related variables such as education, and indirectly, for health behaviors through proxies such as BMI and cigarette smoking. Additionally, poor dental health (measured by the number of missing teeth) was included as an indicator of healthcare access and SES,^2^ which also has been linked to lower diet quality among U.S. adults.^3^

Dr. Zhang also highlights the limitation of our study's cross-sectional design in assessing temporal relationships between *H. pylori* infection and MASLD. Please note that we acknowledged this limitation in the Discussion section and noted that the inability to infer causality is a constraint of our study.^1^

Dr. Zhang also raised concerns about the reliance on self-reported data, particularly regarding health and lifestyle variables such as smoking status and diabetes diagnosis. However, it is important to note that our study incorporated objective biomarkers to complement self-reported data, which enhances the validity of our findings. Specifically, for diabetes we included fasting blood glucose, Hemoglobin A1c levels, and documented use of hypoglycemic agents based on the American Diabetes Association definitions,^4^ ensuring a more accurate assessment of diabetes. Similar objective measures were used for assessing liver disease and *H. pylori* seropositivity. Regarding smoking, it is unclear how a complete smoking history would be obtained without asking the study participant.

We agree that future research is warranted to strengthen the evidence reported in our study and to examine causality. However, as we have acknowledged, this study represents an important first step in understanding the relationship between *H. pylori* and MASLD in a large, diverse Hispanic/Latino population with high prevalence of both conditions.

## Contributors

CSA, BIG, KAM conceptualized and drafted the response letter. All authors reviewed and approved the final version.

## Declaration of interests

Dr. Thyagarajan reports funding and grants from NIH during the preparation of this letter. The other authors have no competing interests.
